# Anti-Ulcer Activity of Essential Oil Constituents

**DOI:** 10.3390/molecules19055717

**Published:** 2014-05-05

**Authors:** Francisco de Assis Oliveira, Luciana Nalone Andrade, Élida Batista Vieira de Sousa, Damião Pergentino de Sousa

**Affiliations:** 1 Universidade Federal do Piauí, Coordenação do Curso de Farmácia, Teresina, PI 64049-550, Brazil; 2 Universidade Federal de Sergipe, Departamento de Farmácia, São Cristóvão, SE 49100-000, Brazil; 3 Universidade Federal da Paraíba, Departamento de Ciências Farmacêuticas, João Pessoa, PB 58051-970, Brazil

**Keywords:** ulcer, gastroprotective, essential oil, monoterpene, phenylpropanoid, anti-inflammatory, antiulcerogenic, natural products

## Abstract

Essential oils have attracted considerable worldwide attention over the last few decades. These natural products have wide-ranging pharmacological activities and biotechnological applications. Faced with the need to find new anti-ulcer agents and the great effort on the development of drugs for the treatment of ulcers, in this review, the anti-ulcer activities of 21 bioactive compounds found in essential oils are discussed.

## 1. Introduction

Peptic ulcer disease is a chronic pathology that affects millions of people worldwide. It is believed that 10% of the population will develop this condition at some point in their lives [1]. Peptic ulcers are usually classified by their anatomic location, such as gastric or duodenal ulcers, and increased gastric acid is the main cause. There is a strong association between *H. pylori* infection and duodenal ulcers. *H. pylori* causes an inflammatory response in the gastric mucosa, with increased production of cytokines [2] and influx of neutrophils and macrophages into the gastric mucosa with release of leukotrienes (LT) and reactive oxygen species, which makes the defense of the mucosa and stimulates of ulcer formation process [3].

The disease process of peptic ulcers is multifactorial based on etiology and risk factors. Ulcers result from an imbalance between aggressive factors, including hydrochloric acid; *Helicobacter pylori* infection; excessive intake of anti-inflammatory drugs, alcohol, pepsin, and reactive oxygen species; and cytoprotective factors, which include mucus, bicarbonate, prostaglandins, blood flow, and cellular repair, as well as enzymatic and non-enzymatic antioxidants [4,5,6].

Currently, the goals of treatment of peptic ulcer are based on pain relief, heal the ulcer and prevent recurrence of the ulcer [7]. Thus, gastric ulcer treatment options include antacids (aluminum hydroxide and magnesium trisilicate), cytoprotective agents (sucralfate and the prostaglandin analogue misoprostol), muscarinic antagonists (pirenzepine), antimicrobial agents for eradication of *H. pylori* (amoxicillin and clarithromycin), H2 receptor antagonists (cimetidine andranitidine), and proton pump inhibitors (omeprazole and lansoprazole) [8,9]. Many adverse effects are associated with the prolonged use of H2 receptor blockers and proton pump inhibitors: hypersensitivity, arrhythmia, impotence, gynecomastia, and hypomagnesemia [10]. Moreover, some of these treatments are expensive [4], and may increase susceptibility to fractures, pneumonia, and gastric cancer [11]. Thus, research to develop new therapeutic agents for gastric ulcer treatment is necessary. In this context, medicinal plants are known to be important resources of bioactive molecules with anti-ulcerogenic potential [12,13]. Several plants have been used in traditional medicine for their anti-ulcerogenic properties. Among medicinal plants, the aromatic species have been used since antiquity because of therapeutic properties that are traditionally attributed to the presence of essential oils [14,15].

Essential oils are complex mixtures of volatile compounds, and are characterized by a strong odor. They comprise mainly of two biosynthetically distinct groups of low-weight molecules: terpenes and phenylpropanoids. Phenylpropane derivatives and aromatic compounds are found less frequently than terpenes [16].

Terpenes found in essential oils are classified as monoterpenes and sesquiterpenes, and these are considered primary classes of secondary metabolites for anti-ulcer activity research [17]. Recently, Rozza and collaborators [18] examined the activity of some essential oils traditionally used in the treatment of gastric ulcers, and considered their families and the part of the plant from which the oils were isolated.

This article reviews studies in the English-language literature on essential oil constituents with anti-ulcer activity. Chemical structures and names of bioactive compounds are provided. The compounds presented in this review were selected with reference to pharmacological action shown in specific experimental models for evaluation of anti-ulcer activity, and/or by complementary studies aimed to elucidate mechanisms of action. The selection of essential oil constituents in the database was performed using various terms, including essential oils, monoterpenes, sesquiterpenes, and phenylpropanoids, as well as names of representative compounds of these groups, and refined with terms relating to anti-ulcer activity, anti-ulcerogenic activity, and ulcers. The search was performed using the Chemical Abstracts Service and Pubmed in November 2013.

## 2. Results and Discussion

### 2.1. Menthol

Menthol [5-methyl-2-(1-methylethyl)cyclohexanol, 2-isopropyl-5-methylcyclohexanol, or *p*-methan-3-ol] is a cyclic monoterpene alcohol that is found as a major constituent in the essential oils of *Mentha canadensis* L (American wild mint) and *Mentha* x *piperita* L (peppermint) [19]. Menthol’s safety profile has been demonstrated by *in vitro* and *in vivo* studies and investigations show a low potential for toxicity in humans [20].

Rozza and collaborators [18] recently showed that oral administration of menthol (50 mg/kg) produced a substantial protective action on the gastric mucosa (88% and 72% against ethanol and indomethacin, respectively). Moreover, the protective mechanism appears to involve increased production of mucus and PGE2, with the involvement of SH compounds and stimulation of K^+^_ATP_ channels, but not the activation of calcium ion channels or the production of nitric oxide. In rats with 4-h pylorus ligation, oral pretreatment with menthol significantly reduced total acid output without modifying its volume. However, intra-duodenal menthol administration diminished the volume of gastric juice, but did not decrease the H^+^ concentration. This study also showed that menthol possesses antiperistaltic activity and no signs of toxicity when orally administered (500 mg/kg) over a period of 14 days.

Considerable attention has been focused on development of ibuprofen prodrugs by selecting various promoieties with the aim of reducing GI toxicity [21,22]. In this direction, [23] have demonstrated improving therapeutic efficacy of racemic ibuprofen (150 mg/kg, p.o.) by retarding gastrointestinal side effects by synthesis and evaluation of ester derivatives of ibuprofen as mutual prodrugs with promoieties like menthol (IME), thymol (ITE) and eugenol (IEE). The results revealed increased anti-inflammatory activity that might be attributed to synergistic effect as ibuprofen conjugates to natural agents. Furthermore the ulcer index shows much reduction in gastric ulceration compared to ibuprofen (2.41 ± 0.27; 0.91 ± 0.15 (IME); 0.83 ± 0.17 (ITE); 1.08 ± 0.15 (IEE)). Finally the studies showed that prodrug approach can be successfully applied in attaining the goal of minimized gastrointestinal toxicity with retention of desired anti-inflammatory activity.

### 2.2. Isopulegol

Isopulegol (*p*-menth-8-en-3-ol) is a monoterpene found in essential oils of various species, such as *Corymbia citriodora* Hill & Johnson [24,25] and *Zanthoxylum schinifolium* Siebold. & Zucc. [26]. This monoterpene belongs to the *p*-menthane family and is used as an intermediate in the preparation of (‒)-menthol [27], and in the manufacture of perfumes, shampoos, and soaps [28]. LD_50_ values in rats treated orally have been reported for isopulegol is 1.03 g/kg bw; [29] and shows no toxicity [30] Silva and collaborators [31], demonstrated that isopulegol (100 and 200 mg/kg) significantly reduced gastric lesions induced by absolute ethanol in mice. Acute administration of ethanol to rodents produces gastric mucosal damage that involves intracellular oxidative stress, inhibition of prostaglandins, intracellular thiol groups, and microcirculation disturbances [32,33]. Therefore, the pathogenesis of ethanol-induced lesions is multifactorial, and this model is widely used to study the cytoprotective potential of drugs [32].

Isopulegol prevented the reduction of non-protein sulfhydryl groups in the gastric mucosa and inhibited ethanol-induced histopathological changes. Hemorrhage, edema, infiltration of inflammatory cells, and reduction of endogenous sulfhydryl levels are characteristicsof damage due to the acute administration of ethanol [34,35]. Taken together, these data indicate significant protective effects of isopulegol, and suggest antioxidant activity.

The roles of prostaglandins, nitric oxide, and ATP-sensitive K^+^ channels (K_ATP_ channels) in the cytoprotective effects of isopulegol were evaluated in the ethanol-induced lesion model after pretreatment of mice with indomethacin, a non-selective cyclooxygenase inhibitor. Indomethacin, an NSAID, caused gastric lesions by the inhibition of prostaglandin biosynthesis, leading to a decrease in gastroprotection, increase in acid secretion, and exacerbation of ulcers [36]. Prostaglandins are responsible for maintaining the integrity of the gastric mucosa by stimulating mucus and bicarbonate secretion [37], and nitric oxide appears to be a major regulator of blood flow and gastric microcirculation [38]. Endogenous prostaglandins act as activators of K_ATP_ channels, and this mechanism, at least in part, mediates gastroprotection in rats [39].

These results show that prostaglandins and potassium channels, but not nitric oxide, participate in the gastroprotective effect of isopulegol, and suggest that isopulegol acts as a cytoprotective agent.

### 2.3. Limonene

d-Limonene [the *R*-(+) isomer] is a monoterpene found in the essential oils of many plants, such as *Artemisia dracunculus* L [40], *Ziziphora taurica* subsp. *cleonioides* P.H. Davis [41], and species of *Protium* such as *P. icicariba* (DC.) Marchand [42] and *P. heptaphyllum* (Aubl.) Marchand [43]. Because of its pleasant citrus fragrance, d-limonene is widely used as a flavoring agent in foods and beverages, and is considered to be a low toxicity ingredient [44].

Studies using pure limonene or essential oils containing *R*-(+)-limonene have demonstrated gastroprotective activity in animal models [45,46,47]. Rozza and collaborators [46] showed that the essential oil of *Citrus lemon* L (250 mg/kg) and limonene (177 mg/kg) exerted marked protection of the gastric mucosa in the ethanol-induced lesion model, and increased expression of the HSP-70 protein, which is associated with cellular adaptive protection processes in response to ethanol ingestion [48], suggesting cytoprotective action. The authors further demonstrated that the essential oil of *C. lemon* and limonene were able to increase levels of mucus and VIP, which were at least partly responsible for the gastroprotective effect of limonene. Gastric wall mucus protects the stomach from injury, and ethanol VIP acts by modulating the effects of histamine, and acting as an anti-inflammatory and antioxidant agent, preventing the formation of gastric ulcers [49].

It is well documented in the literature that the development of ethanol-induced gastric damage is accompanied by decreases in mucosal sulfhydryl compounds [50]. These compounds are neutralized when they bind to the free radicals that are produced following tissue injury by noxious agents. Regulation of levels of GSH, NO participation, and NP-SH compounds should not be considered gastroprotective mechanisms of the essential oil of *C. lemon* or of limonene. The maintenance of PGE2 levels is part of the gastroprotective mechanism of essential oil of *C. lemon*, but not of limonene, suggesting that other components contribute actively to the gastroprotective effects of the essential oil.

*Helicobacter pylori* is an opportunistic pathogen associated with the pathogenesis of chronic gastritis, peptic ulcer, and gastric cancer, and is a global health concern [51]. Antibiotic drug resistance is a major problem in treatment of *H. pylori* infections [52], and thus the search for safe and effective non-antibiotic agents is essential. Limonene showed activity against *H. pylori* with MIC of 75 mg/mL. A result less than or equal to 100 mg/mL MIC is a satisfactory result for products of a natural origin [53]. Thus, these results show potential for the development of limonene as a new treatment for management of *H. pylori* infection.

*Citrus aurantium*, popularly known as bitter orange, is traditionally used in gastrointestinal disorders and rheumatism [54]. Limonene is the main constituent of the essential oil from the peel of the bitter orange fruit [44]. The integrity of the gastric mucosa depends on the balance between aggressive factors and protective mechanisms. One of the current approaches to the treatment of peptic ulcer disease is the inhibition of acid secretion and the promotion of cytoprotection or gastroprotection (participation of endogenous factors in gastric mucosal protection through mechanisms not associated with inhibition of acid secretion) [38,55,56].

Moraes and collaborators [45] characterized the effects of the essential oil of *C. aurantium* L and limonene in animal models of gastric ulcers. In the ethanol-induced gastric ulcer and NSAID models, both the essential oil of *C. aurantium* and limonene (250 and 245 mg/kg, respectively) exerted gastroprotective effects. This result suggests gastric mucosal protective activity of the essential oil of *C. aurantium* and limonene [57,58]. In the ligated pylorus model, it was observed that neither *C. aurantium* oil nor limonene were able to alter parameters of gastric secretion, suggesting that the gastroprotective effect of these products does not occur as a result of antisecretory action. These are interesting data, because the long-term use of proton pump inhibitors or H2 receptor blockers can cause serious adverse effects [59].

Mucus plays an important role in gastroprotection, maintaining the integrity of mucous membranes and neutralizing acid [60]. The essential oil of *C. aurantium* and limonene were tested in a model of ethanol-induced gastric injury, and results showed an increase in the amount of mucus adherence in animals treated with the essential oil and limonene, which underlies their gastroprotective action. Oxidative stress induces gastric mucosal ischemia, leading to increased production of ROS and lipid peroxidation. In this context, increased levels of SOD play an important role in protection from oxidative stress [61]. Moreover, neither *C. aurantium* oil nor limonene changed levels of GSH or SOD in the ethanol injury model, suggesting that the protective effect exhibited by these products is not related to antioxidant activity, but is instead due to increased production of mucus. This conclusion was confirmed in the indomethacin ulcer model. In this experiment, both *C. aurantium* essential oil and limonene were able to maintain levels of PGE2 similar to those found in the mice treated with vehicle, without modifying basal levels of PGE2. This result implicates the mucus-protective actions of the essential oil of *C. aurantium* and limonene in their gastroprotective effects.

### 2.4. Cineole

Cineole (also known as 1,8-cineole, epoxy-*p*-menthane, or eucalyptol) is the primary monoterpene (80%) found in eucalyptus essential oil, and is also present in essential oils of other genera, including *Artemisia*, *Salvia*, and *Mentha* [62]. Cineole is commonly used as a flavoring agent in the food industry and can be found in numerous cleaning products and cosmetics, including toothpastes, soaps, and creams, where it can be absorbed through the skin [63]. This monoterpene shows very low toxicity, the oral LD50 was 2.48 g/kg body weight in the rat showing very low toxicity. Cineole is used in traditional medicine for the treatment of respiratory tract infections [64]. The gastroprotective activity of cineole was investigated using the absolute ethanol gastric lesion model in rats [24]. Male Wistar rats that were pretreated orally with cineole (50 to 200 mg/kg) showed a significant reduction in lesions, and 200 mg/kg was found to be the most potent dose, with effects comparable to those produced by nordihydroguaiaretic acid (NDGA; 75 mg/kg), a lipoxygenase inhibitor [65].

Lipoxygenase seems to play an important role as a mediator of inflammatory responses in gastric ulcers [66]. Leukotriene LTC is an arachidonic acid metabolite produced by lipoxygenase that produces inflammatory vasoconstriction in the gastric mucosa *in vivo*. Ethanol-induced gastric lesions are accompanied by increased production of LTB4 and LTC4, and various non-specific inhibitors of leukotriene synthesis have been shown to prevent this type of damage in the gastric mucosa [67]. Cineole prevented gastric injuries induced by ethanol (50–200 mg/kg), suggesting inhibition of lipoxygenase. This result confirmed the results of the study by Juergnes and collaborators [68] that demonstrated inhibition of arachidonic acid metabolism and generation of leukotrienes after cineole treatment. Santos and Rao [24] showed that cineole (100 and 200 mg/kg) attenuated the ethanol-induced decrease in gastric non-protein sulfhydryl levels, suggesting an antioxidant effect. The antioxidant activity of cineole was confirmed by Santos and collaborators [69] who showed that cineole (400 mg/kg) restored glutathione (GSH) levels and reduced myeloperoxidase (a biochemical marker of neutrophil infiltration in inflamed tissues) activity in a model of trinitrobenzene sulfonic acid (TNBS)-induced colitis, and exerted anti secretory activity in the ligated pylorus model in rats.

The ligated pylorus model is widely used to study drug effects on gastric acid secretion. Pylorus ligation increases the secretion of hydrochloric acid, leading to self-digestion and destruction of gastric mucosa barrier mucus. These events are associated with the development of lesions and ulcers in the upper gastrointestinal tract, leading to perforation and bleeding [70]. Agents that reduce gastric acid secretion and increase mucus secretion are effective in protecting against ulcers. Cineole increased the volume and acidity of gastric secretion. Together, these data show the potential of cineole as an agent for the treatment of gastric ulcers. However, the effect of cineole in the ethanol injury model seems to be independent of its effect on gastric secretion and gastric mucus, because gastroprotection was observed at almost all doses.

### 2.5. Thymoquinone

Thymoquinone (TQ), the primary constituent of volatile oil from *Nigela sativa* seeds, has been reported to inhibit the generation of eicosanoids in leukocytes by inhibiting lipid peroxidation in phospholipid liposomes in the brain [71], and has also shown antioxidant activity [72]. Evidence indicates that TQ has a protective role against gastric ulcers. El-Abhar and collaborators [73] demonstrated that TQ increased levels of SOD and glutathione, and reduced generation of leukotrienes, which were related to its gastroprotective effects in a rat model of reperfusion ischemia. Ischemia and reperfusion are known to induce gastric lesions predominantly because of excessive formation of reactive oxygen metabolites, adhesion of neutrophils to endothelial cells, and microvascular dysfunction [74]. From this study, it may be concluded that the gastroprotective action of TQ can be attributed, at least in part, to its reactive oxygen species-scavenging ability.

Following the study of El-Abhar and collaborators [73], Arslan and collaborators [75] investigated the antioxidant capacity of TQ, and showed that decreased GSH and increased MDA levels in the ethanol-induced ulcer model were reversed by TQ (20 mg/kg), but that the compound produced only a minor non-significant decrease in SOD activity. Kanter and collaborators [76] also investigated the antioxidant effects of TQ in a rat mode of acute ethanol-induced gastric mucosal lesions, and found that TQ (10 mg/kg) mitigated most of the biochemical adverse effects induced by alcohol in the gastric mucosa, such as effects on MDA, SOD, and GSH. However, these effects were observed to a lesser extent than with the essential oil of *N. sativa* L.

The gastroprotective effects of TQ might be mediated by an antihistamine effect. Ohta and collaborators [77] showed that acutely released endogenous serotonin contributed to formation of gastric mucosal lesions in rats treated once with mast cell degranulator C48/80, while endogenous histamine release contributed to lesion progression [78]. In fact, antihistamine action has been shown to be an important preventive mechanism against gastric injury produced by ethanol [79].

Kanter and collaborators [80] showed that gastric tissue histamine levels and MPO activity were increased in ethanol-treated rats, and treatment with *N. sativa* (500 mg/kg) or TQ (10 mg/kg) reversed this effect. These results are in agreement with those obtained by Chakravarty [81], who showed that *N. sativa* and nigellon (polymer of carbonyl TQ isolated from *N. sativa*) inhibited histamine release from rat peritoneal mast cells *in vitro*. This effect might be related to a reduction in c-AMP level, which may be owing to inhibition of adenylate cyclase or stimulation of phosphodiesterase activity. Taken together, these results suggest that *N. sativa* and TQ promote gastroprotection through antioxidant and antihistaminic effects.

Magdy and collaborators [82] recently investigated the mechanisms of TQ gastroprotection by studying its effect on gastric mucosal proton pump (H^+^/K^+^-ATPase) activity, gastric juice components, mucin level, nitric oxide metabolites, and neutrophil infiltration using the hypoxia/reoxygenation model. TQ-treated (20 mg/kg) rats showed marked protection against ischemia/reperfusion insult (I/R). TQ decreased several noxious factors such as acid output, lipid peroxide level, and neutrophil infiltration. TQ-mediated decreases in acid levels were attributed to its ability to normalize proton pump activity in parietal cells and/or antihistaminic activity. TQ also reduced the peptic activity of gastric juice, which may have involved inhibition of histamine release and/or decreased gastric acidity, which attenuated the activation of pepsinogens in the gastric juice. TQ also reduced neutrophil invasion, as evidenced by the decreased activity of MPO and reduced I/R-induced lipid peroxidation, and depleted GSH, SOD, and NO; this finding was in agreement with that of a previous study [64]. The free radical-scavenging/antioxidant properties of TQ may be partly attributable to its metabolite dihydrothymoquinone [83]. Regarding their toxicity, the reported TQ LD_50_ in rats after intraperitoneal injection was 57.5 mg/kg and after oral ingestion was 794.3 mg/kg [84] what justifies the low doses found in the gastroprotective effect of this compound.

### 2.6. Carvacrol

Carvacrol (2-methyl-5-isopropylphenol) is a phenolic monoterpene present in essential oils produced by numerous aromatic plants, such as *Nigella sativa* L, *Origanum vulgare* L, and *Thymus vulgaris* L [85,86]. Carvacrol showed low toxicity in rats [87] and is recognized as a safe food additive and flavoring agent in beverages and chewing gum [88]. Carvacrol shows anti-inflammatory activity *in vitro* [89], as well as antinociceptive and antioxidant activities [90]. A recent study conducted by Silva and collaborators [91] evaluated the anti-inflammatory and anti-ulcer activities of carvacrol. Carvacrol (50 mg/kg) reduced paw edema in several models of inflammation (dextran, histamine, and substance P), and ear edema induced by 12-*O-*tetradecanoylphorbol acetate and arachidonic acid in mice. Furthermore, carvacrol showed healing activity on gastric lesions induced by acetic acid after 14 days of treatment. Acetic acid-induced damage in the gastric ulcer model represents human peptic ulcer disease [92], and the degree of injury is regulated by multiple factors, such as prostaglandins, growth factors, adherent mucus, nitric oxide, and cytokines [93]. Carvacrol showed healing activity on gastric lesions induced by acetic acid at doses of 25, 50, and 100 mg/kg, which produced lesion reductions of 60%, 91%, and 81%, respectively. These effects of carvacrol may result from the inhibition of inflammatory mediators [94].

Oliveira and collaborators [95] evaluated the mechanisms through which carvacrol exerted gastroprotective effects in rodents, and showed that carvacrol (25 mg/kg) showed gastroprotective effects in several mouse and rat models of gastric lesions (absolute ethanol, ethanol-acidified, ischemia and reperfusion, nonsteroidal anti-inflammatory drugs), which were mediated by endogenous prostaglandins, increased mucus production, K_ATP_ channel opening, and NO and antioxidant properties. Carvacrol also inhibited COX-2 [96] antioxidant activity and pro-inflammatory cytokine TNF-α production [90]. Therefore, these effects may be important mechanisms through which carvacrol produces anti-inflammatory and ulcer healing effects.

### 2.7. α-Terpineol

α-Terpineol is a volatile monoterpene alcohol with a slightly sweet odor that is found in the oils of several herbs, such as *Pandanus odoratissimus* L and *Carthamus tinctorius* L [97,98]. This monoterpenoid is generally recognized as safe, because the 50% oral lethal dose in rats and mice is over 5,000 mg/kg [99]. Pharmacological studies have shown that α-terpineol possesses anticonvulsant [100], antinociceptive [101], and hypotensive [102] effects. Souza and collaborators [103] showed that α-terpineol showed gastroprotective activity against ethanol-induced ulcer sat doses of 10, 30, and 50 mg/kg, and also reduced gastric lesions induced by indomethacin at doses of 30 and 50 mg/kg. Pretreatment with indomethacin (10 mg/kg) did not inhibit the gastroprotective action of α-terpineol (50 mg/kg) on ethanol-induced lesions, suggesting that the gastroprotective action of α-terpineol does not involve increased prostaglandin synthesis. Gastric volume, pH, and proton concentration values were not altered by α-terpineol after pylorus ligation, indicating that its gastroprotective action does not involve inhibition of gastric acid secretion. Therefore, the observed gastroprotective effects of α-terpineol probably involve the participation of cytoprotective mechanisms.

### 2.8. Terpinen-4-ol

Terpinen-4-ol is a cyclic monoterpene that is widely distributed in aromatic plants, and found in essential oil from *Zingiber montanum* Link ex Dietr. [104], *Zingiber cassumunar* Link ex Dietr.[105], *Malaleuca alternifolia* Cheel [106], *Origanum majorana* L [107], as well as other species. Concerning the toxicity of terpinen-4-ol, there is interest with respect to observed nephrotoxic effects of oils of which terpinen-4-ol is the main constituent. However, the available data do not give clear evidence for such an effect [108].

Terpinen-4-ol has many pharmacological activities, including anti-inflammation [109] and hypotension effects [110]. Matsunaga and collaborators [111] showed that essential oil from the leaves of *Cryptomeria japonica* D. Don inhibited gastric ulcers induced by several mediators, including HCl/ethanol, HCl/aspirin, water-immersion stress, and pylorus ligation. Among the major components of the essential oil of *C. japonica*, terpinen-4-ol was found to be the most potent in reducing gastric acid secretion and pepsin activity.

### 2.9. Epoxycarvone

Epoxycarvone (EC) is a monocyclic monoterpene found in the essential oils of some plants such as *Carum carvi* L [112] and *Kaempferia galangal* L [113]. In the study of acute toxicity, the LD_50_ calculated was 923 mg/kg with confidence interval of 820 to 1037 mg/kg, suggesting low toxic effect [114].

Previous studies have demonstrated that EC shows activity in the central nervous system [88], anticonvulsant action [115] and antibacterial effects against *Staphylococcus aureus* and *Candida albicans* [116]. Rocha and collaborators [117] showed that intraperitoneal administration of epoxy-carvone (300 mg/kg) produced a significant antinociceptive effect in the acetic acid-induced abdominal writhing test and formalin-induced nociception (in the first and second phases) in mice. EC also inhibited the increased vascular permeability provoked by acetic acid, demonstrating interference with acute inflammatory processes.

To evaluate the gastroprotective activity of epoxycarvone, Siqueira and collaborators [118] studied its effect in models of ulcers induced by ethanol and indomethacin in rats. Epoxycarvone (10 to 50 mg/kg) showed gastroprotective effects in both ulcer models. However, these effects did not involve antisecretory activity or increased synthesis of nitric oxide and prostaglandins.

### 2.10. Elemol

Elemol, or cyclohexanemethanol, is a nontoxic sesquiterpene (Belsito *et al.* [119]) used as a fragrance ingredient in decorative cosmetics, fine fragrances, shampoos, and soaps, as well as in non-cosmetic products such as household cleaners and detergents [120]. Elemol is a major constituent of the essential oil of *Maclura pomifera* which is used as an insect repellent [121]. Elemol is present in the oil composition of *Juniperus* s*altuaria* Rehd & Wils and *J. squamata* var. *fargesii* Redh. And Wils, which possess antifungal activity [122].

According to Matsunaga and collaborators [111], essential oil from the leaves of *Cryptomeria japonica* D. Don (EOCJ) shows anti-ulcer activity, and significantly decreases gastric lesions in the HCl/ethanol, HCl/aspirin, water-immersion stress, and pylorus ligation models. Compounds with anti-ulcer activity were separated from EOCJ by use of distillation and chromatography, and terpinen-4-ol and elemol were isolated as active compounds.

### 2.11. Nerolidol

Nerolidol (3,7,11-trimethyl-1,6,10-dodecatrien-3-ol) is a natural aliphatic sesquiterpene alcohol that is an important component of essential oils from many plants [123,124], and is approved by the U.S. Food and Drug Administration as a food flavoring agent [43].

Klopell and collaborators [125] studied the anti-ulcer activity of *Baccharis dracunculifolia* DC essential oil and its main constituent nerolidol using the ethanol-induced model of acute gastric lesions. Ulcerative lesions in the indomethacin- and stress-induced ulcer models were reduced by oral administration of the essential oil of *B. dracunculifolia* (50–500 mg/kg) or nerolidol (50–500 mg/kg). In agreement with previous reports, Massignani and collaborators [126] reported that the essential oil obtained from the aerial parts of *B. dracunculifolia* significantly diminished (at doses of 50 mg/kg and 500 mg/kg) total lesion area, lesion occurrence, and the volume and acidity of gastric juice in the ethanol-, indomethacin-, and stress-induced ulcer models, and significantly increased gastric pH in the pylorus ligation model. Phytochemical analyses carried out on *B. dracunculifolia* essential oil indicated that nerolidol (23.58%) could be the constituent primarily responsible for its anti-ulcer activity.

### 2.12. α-Bisabolol

(‒)-α-Bisabolol, is a natural, unsaturated, monocyclic sesquiterpene alcohol found in the oils of *Matricaria chamomilla* L, *Vanillosmopsis erythropappa* Schult. Bip., and other plants [127]. The oral acute toxicity (LD_50_) of (‒)-α-bisabolol in mice was 5.1 mL/kg body weight and 14.9 mL/kg for ras [128]. From this data it is possible to conclude that the bisabolol is safe.

This sesquiterpenoid compound is used as a fragrance ingredient in decorative cosmetics, fine fragrances, shampoos, toilet soaps, and household cleaners [28]. (‒)-α-Bisabolol and its oxidized metabolites are used as marker compounds for distinguishing different chemotypes. These compounds produce the aroma of the essential oil of *M. chamomilla* and contribute to its therapeutic properties [129]. Many authors have suggested that (‒)-α-bisabolol possesses a variety of biological activities, including anti-fungal [130], antinociception [131] and antitumor effects [132].

Leite and collaborators [131] showed gastroprotective effects exerted by essential oil from the bark of *Vanillosmopsis arborea* Baker, of which (‒)-α-bisabolol is a major component, at doses of 200 and 400 mg/kgin a model of ethanol-induced lesions in mice. Subsequently, Bezerra and collaborators [133] demonstrated that animals orally treated with *Matricaria recutita* L extract (200 and 400 mg/kg) or (‒)-α-bisabolol (50 and 100 mg/kg) were protected from damage caused by ethanol with effectiveness of 78%–96%. In mechanistic studies, the gastroprotective effect of (‒)-α-bisabolol (100 mg/kg) was reversed when the animals were pretreated with glibenclamide (a K_ATP_ channel blocker), suggesting a role of endogenous prostaglandins in its gastroprotective activity [39].

Aiming to expand gastroprotective studies with (‒)-α-bisabolol, Rocha and collaborators [134] investigated the anti-ulcer activity of this terpene in the indomethacin- and alcohol-induced ulcer model in mice, and the mechanisms involved in this effect. (−)-α-bisabolol (100 and 200 mg/kg) was effective in protecting the gastric mucosa against lesions induced by ethanol and indomethacin when compared to the control group. When animals in the ethanol injury model were pretreated with indomethacin, glibenclamide, or L-NAME (a non-selective competitive inhibitor of NOS), there was no change in the effect produced by (‒)-α-bisabolol (200 mg/kg), suggesting that this effect was not mediated by prostaglandins, nitric oxide, or K_ATP_.

The literature reports that endogenous non-protein sulfhydryl (NP-SH) compounds are important mediators of gastric mucosa protection in models of gastric lesions induced by ethanol and indomethacin [50,135]. (‒)-α-Bisabolol (200 mg/kg) prevented the depletion of GSH promoted by ethanol and indomethacin, which may be associated with its antioxidant properties. ROS have also been shown to play a critical role in gastric ulceration induced by ethanol and NSAIDs. Therefore, enzymatic and non-enzymatic antioxidant defenses play an important role in the prevention of gastric damage. In this context, Rocha and collaborators [136] investigated the antioxidant effect of (‒)-α-bisabolol using an ethanol-induced injury model to better characterize its antioxidant mechanisms. MDA is the final product of lipid peroxidation and is used to determine lipid peroxidation levels in tissues [137]. Ethanol causes necrotic lesions of the gastric mucosa in a multifactorial manner. These effects are probably due to biological actions, such as lipid peroxidation, free radical formation, oxidative stress, and changes in permeability [138]. Organisms have enzymatic and non-enzymatic defenses against ROS-induced lipid peroxidation, including GSH, SOD, and GSH-px [139]. Ethanol has been shown to markedly increase MDA level, an index of lipid peroxidation, which is accompanied by a decrease in GSH, SOD, and GSH-px, which are endogenous antioxidants [140]. (‒)-α-Bisabolol prevented the increase in MDA induced by ethanol, increased SOD activity, and reduced CAT activity. However, this activity does not seem to involve the participation of nitric oxide. Recently, (−)-α-bisabolol showed anti-inflammatory and antinociceptive activity by decreasing leukocyte migration, protein extravasation, and TNF-α levels in a model of carrageenan-induced peritonitis in mice [118]. These findings support the gastroprotective activity of (‒)-α-bisabolol, because ethanol-induced lesions have the characteristic of inducing inflammatory neutrophil migration in gastric lesions, leading to activation of the pro-inflammatory cascade [141].

### 2.13. Anethole

Anethole (1-methoxy-4-(1-propenyl)-benzene) is an aromatic compound largely used in industry as a flavor agent in food and alcoholic beverages. Anethole also possesses anti-inflammatory [142] and anesthetic activities [143]. Freire and collaborators [144] determined the antioxidant, anti-inflammatory, and gastroprotective activity of anethole and hydroxylated derivatives. In the DPPH model, which assesses the inhibitory activity of free radicals generated during lipid peroxidation [145], as well as the acetic acid-induced vascular permeability model, anethole derivatives (300 mg/kg) showed antioxidant and anti-inflammatory activity. These results show the correlation between the antioxidant and anti-inflammatory activities of anethole derivatives, in addition to the presence of phenolic groups that act as antioxidants, due to their ability to scavenge free radicals associated with various human diseases [142]. To assess the gastroprotective effects of anethole and derivatives, these compounds were investigated in the ethanol-induced ulcer model in mice. Anethole (300 mg/kg) increased the level of gastric mucus, while derivatives (300 mg/kg) showed gastroprotective activity without affecting mucus secretion. This result indicates that the double bond and polar group in the side chain of anethole increase gastroprotective activity, but reduce activity on mucus secretion [144].

*Croton zehntneri* Pax et Hoff (*Euphorbiaceae*) is a bush native to northeast Brazil, where it is commonly known as “canela de cunhã” and “canela de cheiro” [146]. Preliminary findings have demonstrated that the leaves of *C. zehntneri* have a rich essential oil content, and one of its principal characteristics is a strong and pleasant odor reminiscent of anise and clove [146]. Anethole is a principal constituent of the essential oil of *Croton zehntneri* (EOCZ), which is used therapeutically in popular folk medicine in a variety of situations, including the relief of pain and anxiety, and for the treatment of gastrointestinal disturbances [147]. Coelho-de-Souza [148] demonstrated that oral treatment with EOCZ and its main constituent anethole at doses of 30, 100, and 300 mg/kg, caused gastroprotection against ethanol- and indomethacin-induced gastric damage. Moreover, neither EOCZ nor anethole reduced the lesion index in cold-restraint stress-induced ulcers in rats. These studies also revealed that pretreatment with EOCZ and anethole (30 and 300 mg/kg) significantly increased mucus production by the gastric mucosa in the ethanol-induced ulcer model. The mechanism of gastroprotection of EOCZ and anethole, at the same doses, seemed not to be related to effective participation of endogenous sulfhydryl groups or on antisecretory effects in pylorus-ligated rats.

Regarding to its toxicity, anethole was examined in some sub-chronic toxicity studies in rats. The only effects observed were slight hepatic changes in the high dose groups (500–700 mg/kg body weight) [149,150]. These observations have been corroborated by [151] that investigated the effect of anethole in a chronic feeding study carried out in rats at concentrations of 0%, 0.25%, 0.5% and 1% for 117–121 wk. The results showed that anethole does not constitute a significant carcinogenic risk to man [151].

### 2.14. Eugenol

Eugenol is a phenylpropanoid and a major constituent of the essential oil of *Syzygium aromaticum* L, a plant commonly known as “cravo da india” that possesses several medicinal properties, including use as an antiseptic and analgesic in dental care, where the undiluted oil may be rubbed on the gums to treat toothache [152]. The LD_50_ for eugenol, administered to rats by oral route was estimated to be 1.93 g/kg [153] and to mice was 3,000 mg/kg [154]. Taken together these data show that this compound has low toxicity and may serve to guide to safe dosage in man.

Eugenol inhibits allergic inflammatory responses *in vitro* and *in vivo* in animals [155], and has been found to have significant anti-inflammatory activity in rats [156].

Santin and collaborators [157] investigated the gastroprotective effects of eugenol in different animal models, and demonstrated that eugenol (100 and 250 mg/kg) reduced ethanol-induced gastric lesions (72% and 95%, respectively) compared to the control group. In the indomethacin-induced ulcer model, eugenol (50 and 250 mg/kg) significantly reduced incidence of ulcers. Furthermore, eugenol (250 mg/kg) did not affect secretion or gastric parameters (volume of gastric juice and acidity) in the pylorus ligation model, which suggests that the gastroprotective action of this substance is not related to antisecretory effects. Eugenol (100 and 250 mg/kg) increased the production of gastric mucus compared to the control group. The most likely mechanism of eugenol gastroprotection is related to factors that increase mucus production and barrier resistance [158]. In an attempt to determine the gastroprotective mechanism of action of eugenol, we assessed the involvement of endogenous nitric oxide and participation of endogenous sulfhydryl, but results showed that the effect of eugenol is not associated with nitric oxide activity or increased endogenous SH. Capasso and collaborators [159] showed that eugenol exhibited dose-dependent gastroprotection (10 to 100 mg/kg) in models of ethanol-induced injury, and by PAF, a derived from platelet membrane, involved in pathophysiology of different pathological processes, such as gastric ulcer [160] and associated with changes in gastric mucosa of rats treated with ethanol [161].

In an attempt to identify the gastroprotective mechanism of eugenol, Morsy and collaborators [162] evaluated the effect of this phenylpropanoid in the indomethacin-induced gastric ulcer model in rats. The inhibition of prostaglandins by NSAIDs induces neutrophil infiltration, imbalances nitric oxide concentrations [163], increases lipid peroxidation with increased production of reactive oxygen species, and decreases the activity of glutathione peroxidase [164]. Morsy and collaborators [162] showed that pretreatment with eugenol (100 mg/kg)in the indomethacin-induced ulcer model in rats reduced gastric ulcers, gastric acid secretion, and pepsin activity, and increased the concentration of gastric mucin. Endogenous gastric pepsin has an important role in the pathogenesis of gastric ulcers; the accumulation of gastric acid and pepsin leads to self-digestion of the gastric mucosa [165]. Mucin is synthesized and secreted immediately after gastric mucosal damage induced by drugs and parasites, and plays a prominent role in the recovery of injured gastric mucosa [166]. The effects of eugenol may be due to its antisecretory properties and to opening of K_ATP_ channels. Eugenol also reduced levels of MDA and nitrite, and prevented the depletion of reduced glutathione, indicating antioxidant action.

Confirming previous findings, Jung and collaborators [167] demonstrates that eugenol isolated from *Cinnamomum cassia* Nees ex Blume showed antioxidant activity *in vitro* in the DPPH model. Using ascorbic acid (IC_50_ < 1 μg/mL) as a reference value, eugenol showed significant antioxidant activity (IC_50_ < 9 μg/mL). It is known that damage to gastric cells in acute and chronic inflammation is due to the toxicity of reactive oxygen species generated in the stomach, so ROS play an important role in the progression of gastric ulcers. Eugenol also demonstrated cytotoxic effects on *H. pylori*, with inhibition at about 8.2 mg/mL, which was comparable to reference drug ampicillin, which showed inhibition at 10 g/mL.

The ethanol induction model of gastric lesions is widely used in the investigation of anti-ulcer drugs. Lesions in this model are associated with increased production of ROS, with consequent increased lipid peroxidation, decreased mucus production, and bicarbonate secretion [168]. The participation of HCl causes severe damage to the gastric mucosa, accelerating the injury process [169]. Mice treated with eugenol showed dose-dependent reduction of lesions, with 65% inhibition at a dose of 100 mg/kg, which was greater than that produced by cimetidine, but eugenol did not alter secretion parameters in the gastric pylorus ligation model in Sprague Dawley rats. The authors also assessed the effect of eugenol on mucus secretion in the ethanol induction model of gastric lesions, and found that eugenol (100 mg/kg) increased the content of mucus (183.5 mg, compared to 173.9 mg for the saline control group). These results show that eugenol has gastroprotective antioxidant activity, neutralizes acid secretion, and partially inhibits *H. pylori*, and is therefore a potential candidate for treatment of gastritis.

### 2.15. 1′S-1′-Acetoxychavicol and 1′S-1′-Acetoxyeugenol Acetate

*Alpinia galanga* (L.) Willd, known as galangal, is widely cultivated in China, India, and Southeast Asia. The rhizomes of galangal are widely used as spice or ginger substitutes for flavoring foods, and in traditional medicine [170]. In chemical studies of *A. galanga*, the principal compound, 1'*S*-1'-acetoxychavicol acetate, was reported to possess various biological activities, among them anti-inflammatory [171], antioxidant [172], and anti-ulcer properties [173]. Matsuda and collaborators [174] described the protective effect of 1'*S*-1'-acetoxychavicol and some related phenylpropanoids from the rhizomes of *A. galangal* on gastric lesions induced by several necrotizing agents, and found that 1'S-1'-acetoxychavicol and 1'*S*-1'-acetoxyeugenol acetate (2.0 mg/kg) inhibited ethanol-induced gastric mucosal lesions. In addition, 1'*S*-1'-acetoxychavicol acetate inhibited lesions induced by 0.6 M HCl and aspirin, but did not show a significant effect on indomethacin-induced gastric lesions or acid output in pylorus-ligated rats at doses of 0.5–5.0 mg/kg. The anti-ulcer effects of 1′*S*-1′-acetoxychavicol acetate were attenuated by pretreatment with indomethacin and *N*-ethylmaleimide (an SH blocker on sulfhydryl compounds), and 1'*S*-1'acetoxychavicol acetate significantly increased glutathione levels ingastric mucosa in rats. These findings indicate a strong participation of endogenous prostaglandins and sulfhydryl compounds in the gastroprotective effect of 1'*S*-1'-acetoxychavicol acetate.

### 2.16. Cinnamaldehyde

Cinnamaldehyde (CA) is a major component of cinnamon, a popular spice that has been used in traditional medicine for its antimicrobial and anti-inflammatory effects, in the treatment of diabetes [175,176], and more recently in preventing lipid peroxidation [177].

Mereto and collaborators [178], using Sprague-Dawley rats found that high doses of CA (500 mg/kg, p.o.) for 14 successive days produced a modest but statistically significant genetic alterations at the chromosomal level in the liver, and suggest that the liver is the preferential target of its undesirable effects.

CA is known to have a wide range of biological properties, such as anti-candidal [179], anti-inflammatory [180,181], and anti-oxidative activities [182], as well as important actions in the cardiovascular system, including vasorelaxation and decreases in blood pressure associated with diabetes [183].

Only one study has shown the gastroprotective activity of cinnamon in mice. Tankam and collaborators [184] found that a cinnamon powder diet (100 mg cinnamon powder per gram of food for 4 weeks) significantly protected mice against ulceration by stress, ethanol, HCl, and oral administration of aspirin, but not against ulceration induced by indomethacin. These findings suggest that regular ingestion of cinnamon offers gastroprotection through a cytoprotective mechanism, and that the active compound of cinnamon powder for gastroprotective activity is probably cinnamaldehyde. This result is consistent with earlier studies which reported that cinnamaldehyde has antiulcer activity [185,186].

### 2.17. Cinnamic Acid

Cinnamic acid (Ci), an active ingredient in cinnamon and propolis (used by honeybees as a hive sealant), has shown a variety of pharmacological properties. Concerning its oral acute toxicity, the LD_50_ values have been reported for 4,454 mg/kg bw in rats [187] and >5,000 mg/kg to mice [188], demonstrating that the oral acute toxicity of these cinnamyl derivatives is extremely low.

Conti and collaborators [189] demonstrated that Ci inhibited TNF-α and IL-10 production by human monocytes by blocking Toll-like receptors (TLR-4), a major class of the pattern-recognition receptors (PRRs) that are present on immune cells, which are involved in the induction of inflammation in intestinal epithelial cells [190]. Ci at a concentration of 1 mM significantly inhibited the formation of advanced glycation end products (AGEs). Furthermore, Ci reduced fructosamine levels, and prevented oxidative protein damage, including effects on protein carbonyl formation. These findings suggest that Ci has an antidiabetic effect [191].

In a study on protection against ulcer and gastritis by *Cinnamomi ramulus* Nees ex Blume (CR, *Cinnamomum cassia*) extract and cinnamic acid, Jung and collaborators [167], demonstrated that CR ethanolic extract showed potent antioxidant activity, acid-neutralizing capacity, and cytotoxicity against *Helicobacter pylori*. Likewise, cinnamic acid (100 mg/kg) significantly inhibited (42.8%) HCl/ethanol-induced gastric lesions and increased mucus content in rats. Ci (IC_50_> 300 µg/mL), which was isolated from CR, exhibited low antioxidant activity *in vitro* and protective effects against gastric damage *in vivo* through stimulation of mucus secretion. Taken together, these data suggest that CR and cinnamic acid show potential as gastroprotective agents.

### 2.18. Citral

Citral is a β-substituted vinyl aldehyde that occurs naturally in several plant species, including myrtle trees, African basil, lemons, limes and oranges [192]. Because of its lemon flavor and odor, citral is used as a flavoring and fragrance agent in foods and cosmetics and is recognized as safe [193].

Work carried out by Ortiz and collaborators [194] shows that the gastric injury produced by naproxen (100 mg/kg, p.o.) was able to produce significant gastric injury by the 3 h time-point. Nevertheless the highest doses of combined naproxen and citral (136.4 and 287.4 mg/kg, p.o., respectively) produced less gastric injury than naproxen alone. These data suggest that the naproxen-citral combination interacts at the systemic level, produces minor gastric damage, and potentially has therapeutic advantages for the clinical treatment of inflammatory pain.

### 2.19. Thymol

Thymol, a monocyclic monoterpene compound isolated from *Thymus vulgaris* L or *Origanum vulgare* spp., has been widely used in the pharmaceutical industry [195]. This substance is listed by the US Food and Drug Administration (US-FDA) as a food additive on the “generally recognized as safe” (GRAS) list and therefore it would be considered nontoxic. Previous studies have demonstrated that thymol has anti-bacterial and anti-inflammatory properties [196,197].

Recently, Dhaneshwar and collaborators [198] developed a co-drug of diacerein with thymol. Diacerein is a symptomatic slow acting disease modifying IL-1b inhibitor, known to possess antiarthritic and moderate anti-inflammatory activity [199]. The data obtained shown that chemical linkage of thymol with diacerein improved its bioavailability. The results of ulcerogenic activity, by using the Rainsford’s cold stress model, revealed that diacerein when directly administered orally, showed higher ulcer index (ulcer index: 6.03 ± 0.15), whereas the association diacerin thimol exhibited lower ulcerogenic potential (4.17 ± 1.03) in rats. In Rainsford’s cold stress model there is involvement of the parietal and zymogen cells than is evident after aspirin treatment alone (which leads to mucosal erosions but not ulceration *per se*)*.* Since the physical stress conditions employed mimic the responses in the stomach following psychological stress, the combined aspirin plus stress treatment may serve as a useful model for studying gastric ulcerogenesis representative of that in humans [200].

### 2.20. Bisabolangelone

Bisabolangelone (BISA), a sesquiterpene found in the species from the Umbelliferae Family. BISA shows anti-ulcer and anti-inflammatory [201,202] and recently, in a molecular studies, Kim and collaborators [203] have shown that BISA suppressed MAPK phosphorylation and nuclear translocation of NF-jB p50/p65 suggesting that BISA works by blocking MAPK and NF-jB signaling, confirming that BISA possesses anti-inflammatory properties.

*Angelica polymorpha* Maxim, is a herbal medicine (Umbelliferae) which was popularly used to treat gastric ulcer [204,205]. Several coumarin, monoterpene, and sesquiterpene such as BISA have been reported as biologically active constituents [206,207,208].

Wang and collaborators (2009) [202], evaluated the anti-ulcer effects of BISA from *A. polymorpha* Maxim in ethanol-induced gastric lesion and pylorus ligation in mouse and rats, respectively. The results showed that BISA (3.8, 7.6 and 15.3 mg/kg body weight) produced a significant reduction of the lesion. The extent of inhibitions for the respective doses employed was 64%, 75% and 82%, respectively. In pylorus ligated model, BISA (3.8 to 15.3 mg/kg) significantly inhibited the activity of H^+^/K^+^-ATPase, reduced the volume of gastric juice, raised the pH value of gastric juice, but had no effect on the activity of the pepsase. Taken all together the findings indicate that BISA operates by inhibiting the activity of the H^+^/K^+^-ATPase, then reducing the secretion of H^+^.

## 3. Conclusions

The literature shows that there is limited clinical data available to support the use of herbs as gastroprotective/antiulcer agents and thus, the studies on efficacy and safety are limited. Despite this, there are several botanical products with potential therapeutic applications because of their high efficacy and low toxicity. This review shows that terpenes and phenylpropanoids present in many essential oils have potential for use in peptic ulcer disease. Among the bioactive constituents of essential oils, there are several chemical classes, such as alcohol, phenol, aldehyde, carboxylic acid, ether, quinone and bifunctional molecules. The different experimental conditions, doses, and animals used in testing, not possible to establish a structure-activity relationship in this review. However, the same feature of these molecules is that they are low molecular weight compounds. The anti-ulcer activity of these natural products can be attributed to several mechanisms, such as free-radical scavenging, inhibition of acid secretion, activity against *H. pylori*, and strengthening of the gastric mucosal barrier.

These findings reinforce the importance and usefulness of constituents of essential oils as promising agents in the management of gastric ulcers, a global disease in which there is high unmet needs related to current treatments in terms of efficacy, safety and low cost.

## Supplementary Materials

Possible mechanisms of action from essential oils constituents with anti-ulcer activity (Figure S1); Essential oil constituents with anti-ulcer activity (Table S1), can be accessed at: http://www.mdpi.com/1420-3049/19/5/5717/s1.

## Abbreviations

c-AMP: Cyclic adenosine monophosphate
CAT: Catalase
COX-2: Cyclo-oxygenase-2
DPPH: 2,2-Diphenyl-1-picrylhydrazyl
GSH: Glutathione
GSH-px: Glutathione peroxidase
H**^+^**/K**^+^**-ATPase: Hydrogen potassium ATPase
HCl: Hydrochloric acid
HSP-70: Heat shock protein 70
IC: Inhibitory concentration
KATP channels: ATP-sensitive potassium channel
L-NAME: N (G)-nitro-L-arginine methyl ester
LTB4: Leukotriene B4
LTC4: Leukotriene C4
MDA: Malondialdehyde
MIC: Minimum inhibitory concentration
NDGA: Nordihydroguaiaretic acid
NO: nitric oxide
NOS: Nitric oxide synthase
NP-SH: Nonprotein sulfhydryls
NSAIDs: Nonsteroidal anti-inflammatory drugs
PAF: Platelet-activating factor
PGE2: Prostaglandin E2
pH: Potential of hydrogen
ROS: Reactive oxygen species
SOD: Superoxide dismutase
TNF-α: Tumor necrosis factoralpha
VIP: Vasoactive intestinal peptide
