# Supplementary material for: Anti-Ulcer Activity of Essential Oil Constituents
Source: Molecules. 2014 May 5;19(5):5717–47. doi: 10.3390/molecules19055717 (PMC6290561; doi:10.3390/molecules19055717)
Supplement: Supplementary file 1 [file molecules-19-05717-s002.pdf]

Supplementary Materials

Table S1. Essential oils constituents with anti-ulcer activity.

**Compound**	**Experimental protocol**	**Antiulcer activity and/or mechanism**	**Animal tested**	**Reference**
Menthol 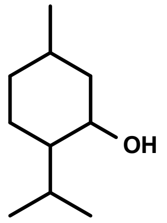	Indomethacin-induced gastric ulcer	Increase PGE2 production<br>Activation of the K^+ATP channels	Wistar rat	[1]
	Ethanol-induced gastric lesions	Increased production of gastric GSH<br>Increase gastric mucus		
	Gastric secretion in 4-h pylorus-ligature	Diminish the H^+ concentration in the gastric juice		
	Ibuprofen- induced gastric ulcer	Cytoprotection		[2]
Isopulegol 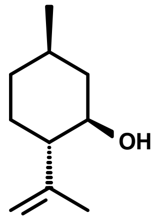	Ethanol-induced gastric lesions in indomethacin-pretreated animals	Increase endogenous prostaglandins<br>K^+ATP channel opening<br>Increase endogenous NO	Swiss mouse	[3]
Epoxy-carvone 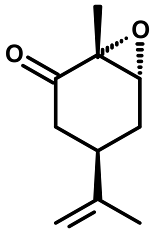	Ethanol and indomethacin-induced gastric ulcer	Gastroprotective activity	Rats	[4]
	Ethanol and indomethacin-induced ulcers	Gastroprotective effects	Rats	[5]
Limonene 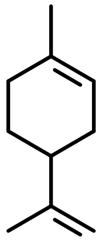	Ethanol- induced gastric lesions	Increased expression of the HSP-70 protein<br>Increase gastric mucus	Wistar rat	[6]
		Increase of VIP		[7]
		Activity against *H. pylori*		[8]
	Indomethacin-induced gastric ulcers	Increase gastric mucus		[9]

Table S1. *Cont.*

Compound	Experimental protocol	Antiulcer activity and/or mechanism	Animal tested	Reference
1,8-Cineole 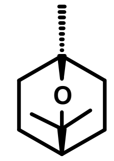	*Ethanol-induced gastric lesions*	Inhibition of lipoxigenase		[10]
		Increased production of gastric GSH	Swiss mouse	[11]
	Gastric secretion in 4-h pylorus-ligature	Reduced the gastric secretory volume and total acid output		[12]
	Induced gastric mucosal injury by ischemia and reperfusion	Increase levels of SOD and GSH		[13]
	Ethanol-induced gastric lesions	Increase GSH content<br>Decreased the MDA level in gastric tissue<br>Decreased the gastric MDA content<br>Increase GSH content<br>Increased the enzymatic activity of SOD		[14]
Thymoquinone 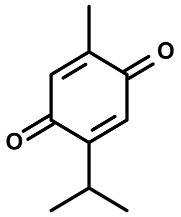	Ethanol-induced gastric lesions	Increase the enzyme activity of gastric GST<br>Reduction in the number of mast cells in the gastric mucosa	Wistar rat	[15]
		Decrease gastric tissue histamine levels		[16]
		Decrease MPO activity		[17]
	Induced gastric mucosal injury by ischemia and reperfusion	Decrease acid concentration and acid output<br>Reduction pepsin content<br>Increased gastric mucin content		[18]
	Induced gastric mucosal injury by ischemia and reperfusion	Low proton pump activity<br>Decrease MPO activity<br>Increase endogenous NO<br>Increase GSH and SOD levels		[19]

Table S1. *Cont.*

Compound	Experimental protocol	Antiulcer activity and/or mechanism	Animal tested	Reference
Carvacrol 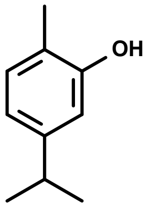	Ethanol -induced gastric lesions<br>Pylorus ligation induced ulceration	Participation of sulfhydryl groups (SH) non-protein<br>Increase catalase activity<br>K^{+}ATP channels Opening<br>Increase endogenous NO<br>Increase in gastric mucus content	Swiss mouse and Wistar rat	[20]
	Pretreatment with ibuprofen on absolute ethanol-induced gastric lesions model	Involvement of endogenous prostaglandin		[21]
Terpinen-4-ol 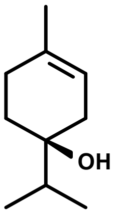	Pylorus ligation induced ulceration	Reduction the volume of gastric juice, acidity and pepsin content	Wistar or Sprague–Dawley rat	[22]
α-Terpineol 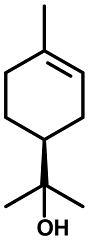	Ethanol and indomethacin-induced gastric ulcer	Gastroprotective activity	Rats	[23]
Elemol 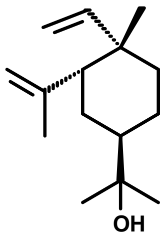	Pylorus ligation induced ulceration	Reduction the volume of gastric juice, acidity and pepsin content	Wistar or Sprague–Dawley rat	[22]
Nerolidol 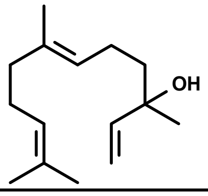	Gastric secretion in 4-h pylorus-ligature	Reduction the volume of gastric juice and the total acidity<br>Increased gastric pH	Wistar rat	[24]

Table S1. *Cont.*

Compound	Experimental protocol	Antiulcer activity and/or mechanism	Animal tested	Reference
1'S-1'-Acetoxychavicol acetate 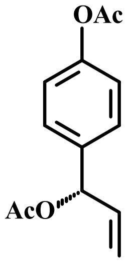	Pretreatment with indomethacin on absolute ethanol-induced gastric lesions model	Involvement of endogenous prostaglandin	Sprague–Dawley rat	[25]
	Gastric lesions induced by ethanol in NEM-pretreated rats	Increase in endogenous SH		
1'S-1'-Acetoxyeugenol acetate 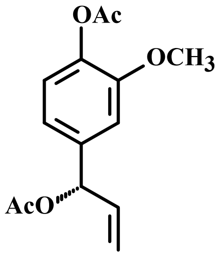	Pretreatment with indomethacin on absolute ethanol-induced gastric lesions model	Involvement of endogenous prostaglandin	Sprague–Dawley rat	[25]
		Increase in endogenous SH		[26]
α-Bisabolol 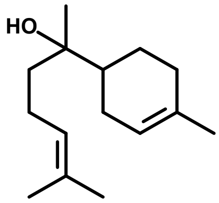	Gastric lesions induced by ethanol in NEM-pretreated rats	Increase SOD activity<br>Decrease the MDA amount in gastric mucosa<br>Reduction the MPO activity in gastric mucosa	Wistar rat	[27]
	Indomethacin-induced gastric ulcers	Increased production of gastric GSH		[28]
Anethole [1-methoxy-4-(1-propenyl)benzene] 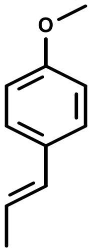		Increased the level of gastric mucus		[29]
	Ethanol- induced gastric lesions	Increase gastric mucus	Swiss mouse	[30]

Table S1. *Cont.*

Compound	Experimental protocol	Antiulcer activity and/or mechanism	Animal tested	Reference
Eugenol (4-allyl-2-methoxyphenol) 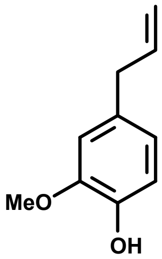	Pylorus ligation induced ulceration	Enhanced gastric mucus	Wistar rat	[31]
	PAF-induced damage	PAF antagonist		[32]
		Reduction gastric acid secretion<br>Decrease pepsin activity<br>Reduction mucin content		[33]
	Indomethacin-induced gastric ulcers	Opening of K^+ATP channel	Sprague-Dawley rat	[34]
		Reduction MDA level<br>Increase endogenous NO<br>Increased production of gastric GSH		[35]
	In vitro assay *Helicobacter pylori*	Supression of *Helicobacter pylori* growth	*Helicobacter pylori* strains	[36]
	Ethanol-induced gastric lesion	Increase gastric mucus	Sprague-Dawley rat	
	Ibuprofen- induced gastric ulcer	Cytoprotection	Wistar rat	[2]
Cinnamaldehyde [(2*E*)-3-phenylprop-2-enal] 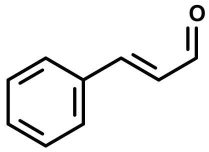	*In vitro* assay *Helicobacter pylori*	Supression of *Helicobacter pylori* growth	*Helicobacter pylori* strains	[37]
Cinnamic acid [(*E*)-3-phenylprop-2-enoic acid] 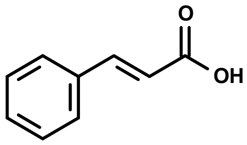	Ethanol -induced gastric lesions	Increase gastric mucus	Sprague–Dawley rat	[36]

Table S1. *Cont.*

Compound	Experimental protocol	Antiulcer activity and/or mechanism	Animal tested	Reference
Citral (= geranial + neral)<br>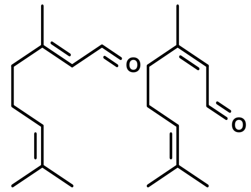<br>(geranial) (neral)	Naproxen –induced gastric injury	Cytoprotection	Wistar rat	[38]
Thymol<br>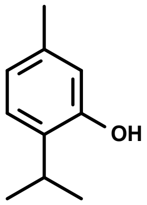	Ibuprofen- induced gastric ulcer	Cytoprotection	Wistar rat	[2]
Bisabolangelone<br>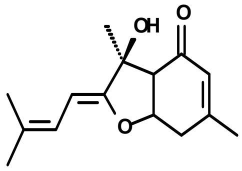	Ethanol -induced gastric lesions<br>Pylorus ligation model	Inhibiting the activity of the H^+/K^+-ATPase; Reducing the H^+ output	Kunming mice<br>Sprague–Dawley rats and Kunming mouse	[39]

Figure S1. Possible mechanisms of action from essential oils constituents with anti-ulcer activity.

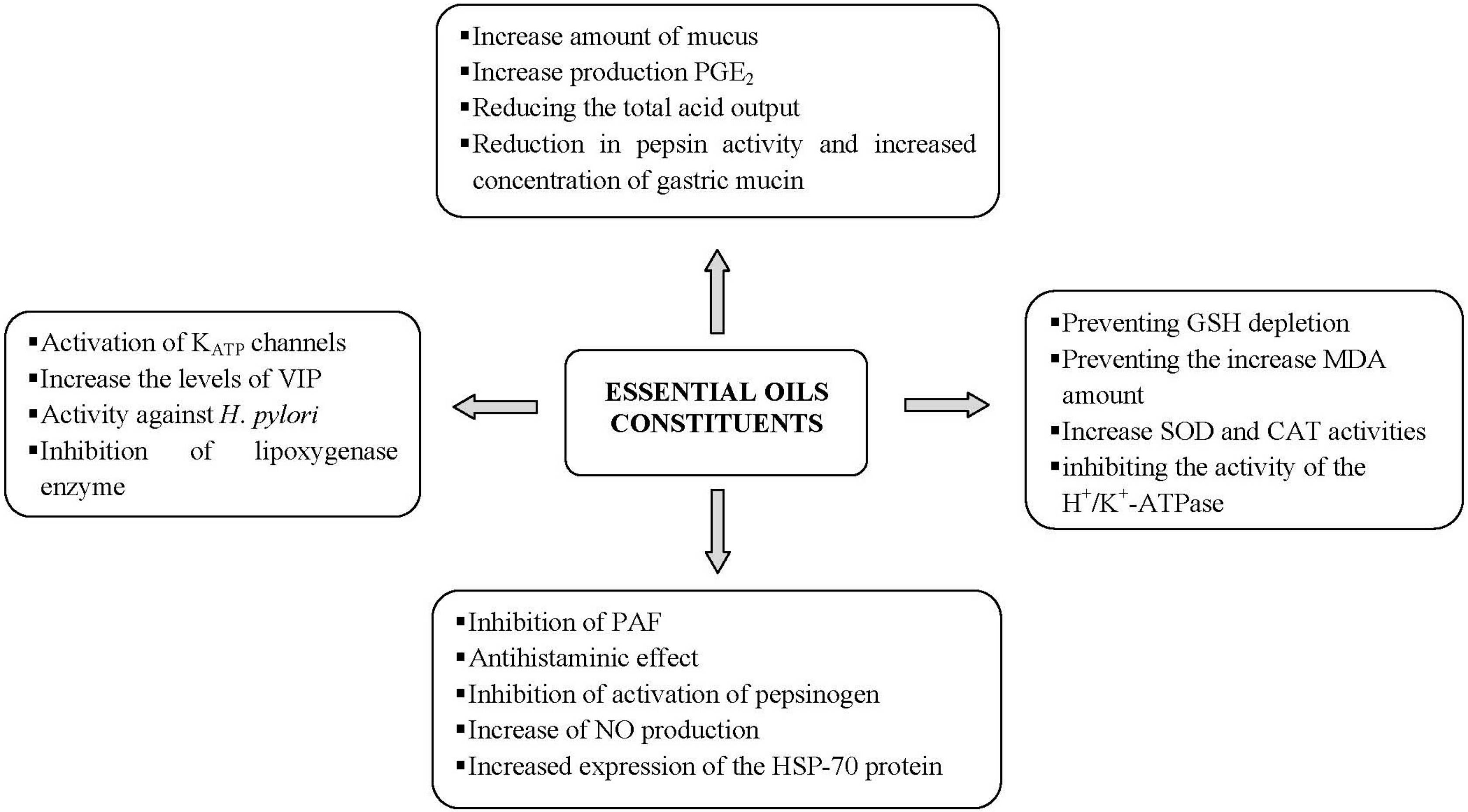

References

1. Rozza, A.L.; Hiruma-Lima, C.A.; Takahira, R.K.; Padovani, C.R.; Pellizzon, C.H. Effect of menthol in experimentally induced ulcers: Pathways of gastroprotection. *Chem. Biol. Interact.* **2013**, *206*, 272–278.
2. Redasani, V.K.; Sanjay B.B. Synthesis and evaluation of mutual prodrugs of ibuprofen with menthol, thymol and eugenol. *Eur. J. Med. Chem.* **2012**, *56*, 134–138.
3. Miller, T.A. Protective effects of prostaglandins against gastric mucosal damage: Current knowledge and proposed mechanisms. *Am. J. Physiol.* **1983**, *245*, 601–623.
4. Rocha, M.L.; Oliveira, L.E.G.; Santos, C.M.P.; De Sousa, D.P.; Almeida, R.N.; Araujo, D.A.M. Antinociceptive and anti-inflammatory effects of the monoterpene α-β-epoxy-carvone in mice. *J. Nat. Med.* **2013**, *67*, 743–749.
5. Siqueira, B.P.J.; Menezes, C.T.; Silva, J.P.; De Sousa, D.P.; Batista, J.S. Antiulcer effect of epoxy-carvone. *Braz. J. Pharmacogn* **2012**, *22*, 144–149.
6. Rozza, A.L.; Moraes, T.M.; Kushima, H.; Tanimoto, A.; Marques, M.O.M.; Bauab, T.M.; Hiruma-Lima, C.A.; Pellizzon, C.H. Gastroprotective mechanisms of *Citrus lemon* (Rutaceae) essential oil and its majority compounds limonene and β-pinene: Involvement of heat-shock protein-70, vasoactive intestinal peptide, glutathione, sulfhydryl compounds, nitric oxide and prostaglandin E2. *Chem. Biol. Interact.* **2011**, *189*, 82–89.
7. Tunçel, N.; Tunçel, M.; Aboul-Enein, H.Y. Effects of the vasoactive intestinal peptide on stress-induced mucosal ulcers and modulation of methylation of histamine in gastric tissue of the rats. *Il Fármaco* **2003**, *58*, 449–454.
8. Mitscher, L.A.; Drake, S.; Gollapudi, S.R.; Okwute, K. Model look at folkloric use of anti-infective agents. *J. Nat. Prod.* **1987**, *50*, 1025–1040.
9. Tariq, M.; Khan, H.A.; Elfaki, I.; Arshaduddin, M.; Al Moutaery, M.; Al Rayes, H.; Al Swailam, R. Gastric antisecretory and antiulcer effects of simvastatin in rats. *J. Gastroenterol. Hepatol.* **2007**, *22*, 2316–2323.
10. Peskar, B.M.; Lange, K.; Hoppe, U; Peskar, B.A. Ethanol stimulates formation of leukotriene C4 in ratgastric mucosa. *Prostaglandins* **1986**, *31*, 283–293.
11. Santos, F.A.; Silva, R.M.; Campos, A.R.; de Araújo, R.P.; Lima Júnior, R.C.; Rao, V.S. 1,8-cineole (eucalyptol), a monoterpene oxide attenuates the colonic damage in rats on acute TNBS-colitis. *Food Chem. Toxicol.* **2004**, *42*, 579–584.
12. Kumar, A.; Singh, V.; Chaudhary, A.K. Gastric antisecretory and antiulcer activities of *Cedrus deodara* (Roxb.) Loud. in Wistar rats. *J. Ethnopharmacol.* **2011**, *134*, 294–297.
13. El-Abhar, H.S.; Abdallah, D.M.; Saleh, S. Gastroprotective activity of *Nigella sativa* oil and its constituent, thymoquinone, against gastric mucosal injury induced by ischaemia/reperfusion in rats. *J. Ethnopharmacol.* **2003**, *84*, 251–258.
14. Kanter, M.; Demir, H.; Karakaya, C.; Ozbek, H. Gastroprotective activity of *Nigella sativa* L oil and its constituent, thymoquinone against acute alcohol-induced gastric mucosal injury in rats. *World J. Gastroenterol.* **2005**, *11*, 6662–6666.

15. Ohta, Y.; Kobayashi, T.; Imai, Y.; Inui, K.; Yoshino, J.; Nakazawa, S. Effect of oral Vitamin E administration on acute gastric mucosal lesion progression in rats treated with compound 48/80, a mast cell degranulator. *Biol. Pharm. Bull.* **2006**, *29*, 675–683.
16. Ohta, Y.; Kobayashi, T.; Ishiguro, I. Participation of xanthine– xanthine oxidase system and neutrophils in development of acute gastric mucosal lesions in rats with a single treatment of compound 48/80, a mast cell degranulator. *Dig. Dis. Sci.* **1999**, *44*, 1865–1874.
17. Kanter, M.; Coskun, O.; Uysal, H. The antioxidative and antihistaminic effect of *Nigella sativa* and its major constituent, thymoquinone on ethanol-induced gastric mucosal damage. *Arch. Toxicol.* **2006**, *80*, 217–224.
18. Magdy, M.A.; Hanan, E.A.; Nabila, E.M. Thymoquinone: Novel gastroprotective mechanisms. *Eur. J. Pharmacol.* **2012**, *697*, 126–131.
19. Mansour, M.A.; Nagi, M.N.; El-Khatib, A.S.; Al-Bekairi, A.M. Effects of thymoquinone on antioxidant enzyme activities, lipid peroxidation and DT-diaphorase in different tissues of mice: A possible mechanism of action. *Cell. Biochem. Funct.* **2002**, *20*, 143–151.
20. Oliveira, I.S.; Silva, F.V.; Viana, A.F.S.C.; Santos, M.R.V.; Quintans-Júnior, L.J.; Martins, M.C.C.; Nunes, P.H.M.; Oliveira, F.A.; Oliveira, R.C.M. Gastroprotective activity of carvacrol on experimentally induced gastric lesions in rodents. *Naunyn-Schmiedeberg's Arch. Pharmacol.* **2012**, *385*, 899–908.
21. Hotta, M.; Nakata, R.; Katsukawa, M.; Hori, K.; Takahashi, S.; Inoue, H. Carvacrol, a component of thyme oil, activates PPARalpha and gamma and suppresses COX-2 expression. *J. Lipid Res.* **2010**, *51*, 132–139.
22. Matsunaga, T.; Hasegawa, C.; Kawasuji, T.; Suzuki, H.; Saito, H.; Sagioka, T.; Takahashi, R.; Tsukamoto, H.; Morikawa, T.; Akiyama, T. Isolation of the antiulcer compound in essential oil from the leaves of *Cryptomeria japonica*. *Biol. Pharm. Bull.* **2000**, *23*, 595–598.
23. Souza, R.H.L.; Cardoso, M.S.P.; Menezes, C.T.; Silva, J.P.; de Sousa, D.P.; Batista, J.S. Gastroprotective activity of α-terpineol in two experimental models of gastric ulcer in rats. *Daru* **2011**, *19*, 277–281.
24. Massignani, J.J.; Lemos, M.; Maistro, E.L.; Schaphauser, H.P.; Jorge, R.F.; Sousa, J.P.B.; Bastos, J.K.; Andrade, S.F. Antiulcerogenic Activity of the Essential Oil of *Baccharis dracunculifolia* on Different Experimental Models in Rats. *Phytother. Res.* **2009**, *23*, 1355–1360.
25. Matsuda, H.; Pongpiriyadacha, Y.; Morikawa, T.; Ochi, M.; Yoshikawa, M. Gastroprotective effects of phenylpropanoids from the rhizomes of *Alpinia galanga* in rats: Structural requirements and mode of action. *Eur. J. Pharmacol.* **2003**, *471*, 59–67.
26. Yoshikawa, T.; Minamiyama, Y.; Ichikawa, H.; Takahashi, S.; Naito, Y.; Kondo, M. Role of lipid peroxidation and antioxidants in gastric mucosal injury induced by the hypoxanthine-x anthine oxidase system in rats. *Free Radic. Bio. Med.* **1997**, *23*, 243–250.
27. Rocha, N.F.M.; Venancio, E.T.; Moura, B.A.; Silva, M.I.G.; Aquino Neto, M.F.; Rios, V.E.R.; Sousa, D.P.; Vasconcelos, S.M.M.; Fonteles, M.M.F.; Sousa, F.C.F. Gastroprotection of (−)-α-bisabolol on acute gastric mucosal lesions in mice: The possible involved pharmacological mechanisms. *Fundam.Clin. Pharmacol.* **2010**, *24*, 63–71.

28. Halici, M.; Odabasoglu, F.; Suleyman, H.; Cakir, A.; Aslan, A.; Bayir, Y. Effects of water extract of Usnea longissima on antioxidant enzyme activity and mucosal damage caused by indomethacin in rats. *Phytomedicine* **2005**, *12*, 656–662.
29. Freire, R.S.; Morais, S.M.; Catunda-Junior, F.E.A.; Pinheiro, D.C.S.N. Synthesis and antioxidant, anti-inflammatory and gastroprotector activities of anethole and related compounds. *Bioorg. Med. Chem.* **2005**, *13*, 4353–4358.
30. Coelho-De-Souza, A.N.; Lahlou, S.; Barreto, J.E.F., Yum, M.E.M.; Oliveira, A.C.; Oliveira, H.D.; Celedonio, N.R.; Feitosa, R.G.R.; Duarte, G.P.; Santos, C.F.; *et al.* Essential oil of *Croton zehntneri* and its major constituent anethole display gastroprotective effect by increasing the surface mucous layer. *Fundam.Clin. Pharmacol.* **2013**, *27*, 288–298.
31. Santin, J.R.; Lemos, M.; Klein-Júnior, L.C.; Machado, I.D.; Costa, P.; Oliveira, A.P.; Tilia, C.; Souza, J.P.; Sousa, J.P.B.; Bastos, J.K.; *et al.* Gastroprotective activity of essential oil of the *Syzygium aromaticum* and its major component eugenol in different animal models. *Naunyn-Schmiedeberg's Arch. Pharmacol*. **2011**, *383*, 149–158.
32. Capasso, R.; Pinto, L.; Vuotto, M.L.; di Carlo, G. Preventive effect of eugenol on PAF and ethanol-induced gastric mucosal damage. *Fitoterapia* **2000**, *71*, 131–137.
33. Morsy, M.A.; Fouad, A.A. Mechanisms of gastroprotective effect of eugenol in indomethacin-induced ulcer in rats. *Phytother. Res.* **2008**, *22*, 1361–1366.
34. Goel, R.K.; Bhattacharya, S.K. Gastroduodenal mucosal defense and protective agents Indian. *J. Exp. Biol.* **1991**, *29*, 701–714.
35. Iwai, T.; Ichikawa, T.; Goso, Y.; Ikezawa, T.; Saegusa, Y.; Okayasu, Y.; Saigenji, K.; Ishihara, K. Effects of indomethacin on the rat small intestinal mucosa: immunohistochemical and biochemical studies using anti-mucin monoclonal antibodies. *J. Gastroenterol.* **2009**, *44*, 277–284.
36. Jung, J.; Lee, J.H.; Bae, K.H.; Jeong, C.S. Anti-gastric actions of eugenol and cinnamic acid isolated from *Cinnamoni ramulus. Yakugaku Zasshi* **2011**, *131*, 1103–1110.
37. Harada, M.; Yano, S. Pharmacological studies on Chinese cinnamon. Effects of cinnamaldehyde on the cardiovascular and digestive systems. *Chem. Pharm. Bull.* **1975**, *23*, 941–947.
38. Ortiz, M.I.; Ramírez-Montiel, M.L.; González-García, M.P.; Ponce-Monter, H.A.; Castañeda-Hernández, G.; Cariño-Cortés, R. The combination of naproxen and citral reduces nociception and gastric damage in rats. *Arch. Pharm. Res*. **2010**, *10*, 1691–1697.
39. Wang, J.; Zhu, L.; Zou, K.; Cheng, F.; Dan, F.; Guo, Z.; Cai, Z.; Yang, J. The antiulcer activities of bisabolangelone from *Angelica polymorpha*. *J. Ethnopharmacol*. **2009**, *123*, 343–346.

© 2014 by the authors; licensee MDPI, Basel, Switzerland. This article is an open access article distributed under the terms and conditions of the Creative Commons Attribution license (http://creativecommons.org/licenses/by/3.0/).
